# Ensino Médico como Interlocutor da Ciência e a Sociedade

**DOI:** 10.36660/abc.20220491

**Published:** 2022-11-09

**Authors:** José Maria Peixoto

**Affiliations:** 1 Universidade José do Rosário Vellano Belo Horizonte MG Brasil Universidade José do Rosário Vellano , Campus Belo Horizonte, Belo Horizonte , MG – Brasil

**Keywords:** Educação Médica, Aprendizagem, Cardiologia/educação, Pesquisa Científica e Desenvolvimento Tecnológico, Docentes de Medicina

A evolução científica observada na área da cardiologia é incontestável. ^1 , 2^ É preciso lembrar, no entanto, que a força propulsora desse desenvolvimento repousa nas *ciências da educação* que, ao mobilizar os indivíduos para o conhecimento, transforma o mundo. Apesar do debate existente sobre a *quais* saberes se relacionam as *ciências da educação* , segundo Kant, ^3^ é por meio delas que os indivíduos alcançam a autonomia e conquistam a liberdade para a vida em sociedade.

A ciência tradicional produz conhecimentos a partir da experimentação e observação, adotando métodos racionais que garantem a validade de seus resultados. Mas, esses pressupostos favorecem uma separação entre o *sujeito e o objeto* de estudo, criando uma cultura técnico-científica que muitas vezes não inclui o pensamento filosófico, condutor do desejo de saber dos indivíduos. ^4^

Há tempos a ciência deixou de ser um método desconectado das questões sociais, passando a servir às necessidades da coletividade. ^5^ A pandemia da COVID-19, iniciada no Brasil em 2020, ^6^ exemplifica este fato e contribuirá para acelerar esse processo. Ao impor desafios à pesquisa científica, que necessitou fornecer respostas rápidas para conter o avanço da doença, a pandemia aproximou a realidade do mundo acadêmico à sociedade. Augusti ^5^ defende ser necessário pensar uma ciência comprometida com a prática social, que incentive a produção de ensino e aprendizagens coletivas. ^5^

Neste ponto, é relevante a contribuição das *ciências da educação* como interlocutora entre as concepções do saber científico e a filosofia, esta última contribuindo para a definição dos fins. Destacam-se, assim, as diferentes funções do processo educacional, que deve se dedicar à profissionalização do indivíduo sem deixar de acolher suas necessidades de crescimento como *pessoa* , nas dimensões afetiva, cultural, ética e política. ^7^

Como pode ser visto, apesar do conhecimento técnico-científico ser relevante para a formação profissional, é insuficiente para atender às demandas do mundo contemporâneo. A Constituição de 1988, ao garantir a saúde como um direito universal, coloca o Sistema Único de Saúde como norteador do modelo assistencial brasileiro, que pressupõe uma mudança do foco predominantemente biomédico da atenção à saúde para um modelo que incorpora a perspectiva biopsicossocial, a interdisciplinaridade e a corresponsabilização. ^8^ Ainda há desafios para a incorporação dessas competências profissionais, que necessita discutir o ensino em saúde e a formação docente em todas as esferas da educação médica. ^8^

Em geral, boa parte da discussão sobre formação médica trata do período de graduação, com pouco debate referente à pós-graduação, em geral desenvolvida em instituições hospitalares e sociedades de especialidades médicas. Considerando-se a importância da “aprendizagem ao longo da vida”, é necessário que o tema da educação médica seja inserido nessas organizações, com o objetivo de fomentar a pesquisa e a profissionalização docente. ^9^

Assim como a atividade profissional médica deve incorporar as melhores evidências científicas para a tomada de decisões, as estratégias instrucionais desenvolvidas pelas instituições responsáveis pela formação médica também deveriam ser orientadas por estudos científicos. As sociedades de cardiologia do mundo inteiro têm definido critérios para a certificação de especialidades e áreas de atuação; no entanto, parte dessas determinações são definidas por consenso de especialistas e não por meio de pesquisa educacional. Nota-se a necessidade de se estender a discussão sobre educação médica à toda a comunidade envolvida em programas de formação médica. ^10^

A educação médica é um campo de investigação científica reconhecida internacionalmente, com ampla comunidade acadêmica dedicada ao assunto, bem como revistas especializadas revisadas por pares. ^11^ Há ainda alguma confusão em relação aos termos Educação Médica e Educação Continuada. Educação Médica é a área do conhecimento dedicada ao desenvolvimento de estudos relacionados ao processo ensino-aprendizagem, enquanto Educação Continuada se dedica à aquisição e atualização de conhecimentos. ^12^ As Sociedades Médicas, em geral, dedicam-se mais às atividades de Educação Continuada, como as desenvolvidas em congressos, seminários, cursos etc.

Apesar da relevância, observa-se que as publicações relacionadas ao ensino raramente são contempladas pelas revistas especializadas em medicina, e sim publicadas em revistas de educação em saúde. Assim, grande parte dessas publicações concentram-se na graduação médica, apesar da formação profissional estender-se para toda a vida.

Alfred et al. ^11^ quantificaram o número de estudos publicados relacionados ao ensino em cardiologia e o número de cardiologistas dedicados à pesquisa em ensino e estimaram o nível de prioridade que as publicações em educação médica possuíam em periódicos de cardiologia. Os resultados foram desapontadores, nas 26 revistas incluídas no estudo, dentre 6645 artigos analisados, apenas quatro eram sobre educação médica. Em 10 revistas de medicina geral e 15 de educação médica incluídas, dentre 6810 artigos analisados, apenas sete tratavam do ensino em cardiologia e todos haviam sido publicados em revistas de educação médica. Importante salientar que dentre esses sete artigos, nenhum dos autores eram cardiologistas e nenhum abordava o ensino na especialização em cardiologia. Em relação ao percentual de cardiologistas dedicados à pesquisa em ensino médico, entre 5584 pesquisadores apenas 2,3% (n=128) eram cardiologistas. Por último, ao avaliarem as definições das missões das revistas de cardiologia, dentre 1036 palavras utilizadas para esse fim, o termo “educacional” foi encontrado apenas na *European Heart Journal.*

Os dados deste estudo chamam a atenção para o pouco interesse que as revistas de cardiologia têm dispensado à educação médica. Entretanto, tanto as Sociedades de Cardiologia, quanto as instituições de treinamento em cardiologia, promovem atividades de ensino e, portanto, há muitos cardiologistas envolvidos com atividades educacionais. Provavelmente, não tem havido estímulos para o desenvolvimento e publicações de estudos relacionado ao ensino médico. De fato, é difícil publicar estudos sobre este tema em periódicos médicos, pela baixa prioridade dada a esses assuntos.

A Sociedade Brasileira de Cardiologia (SBC) é uma entidade científica envolvida na produção de conhecimento médico no Brasil. Ao longo dos anos, vem se destacando pela qualidade de suas produções e inovações assumidas. Seu grupo de colaboradores é constituído por especialistas e pesquisadores com grande expertise, que em geral, desenvolvem além das atividades assistenciais, ações relacionadas à formação médica, tanto na graduação quanto na pós-graduação. Vale ressaltar as relevantes atividades educacionais existentes na SBC, como: a Universidade do Coração, o Curso Auxiliar Preparatório para o Título de Especialista em Cardiologia e os relevantes trabalhos da Comissão Julgadora do Título de Especialista em Cardiologia.

Embora possa parecer que o tema da “Educação Médica” seja novo, provavelmente muitos já praticam atividades exitosas de ensino em seus ambientes de trabalho, que não são compartilhadas com a comunidade acadêmica. Portanto, a criação de um Suplemento dedicado ao Ensino Médico em Cardiologia é uma oportunidade para que professores e pesquisadores possam compartilhar os resultados de seus trabalhos e assim, fomentar uma nova área de conhecimento e atividade profissional para o cardiologista, a Educação Médica.

Mais uma vez, demonstrando sua vocação para ações inovadoras e contemporâneas, a SBC, por meio de sua Diretoria Científica e do corpo editorial dos Arquivos Brasileiros de Cardiologia, representado pelo seu Editor-Chefe, o Prof. Dr. Carlos Eduardo Rochitte, dá mais um passo à frente, e demonstra forte apoio à pesquisa sobre o ensino em cardiologia, materializado nesse suplemento. O espaço para publicações de estudos de alta qualidade sobre o ensino em cardiologia está garantido na ABC Cardiol.

A educação médica possui uma diversidade de temas que podem ser contemplados nos periódicos da SBC como: o ensino do raciocínio clínico, a relação médico-paciente, desenvolvimento do currículo, integração ensino-pesquisa, integração ensino-comunidade, desenvolvimento docente, teorias da aprendizagem, avaliação do processo ensino-aprendizagem, dentre outros.

Pesquisadores envolvidos com o tema da Educação Médica em Cardiologia poderão então compartilhar os resultados de seus trabalhos. Dessa forma, espera-se estimular o desenvolvimento da pesquisa nessa área, fomentar a formação de grupos de estudos em Educação Médica na SBC e contribuir para a profissionalização da docência/preceptoria em cardiologia no Brasil.

Investir em educação e formação docente é um ótimo caminho para se formar um profissional de qualidade, com capacidade para compreender e atender as demandas sociais, objetivando sempre a melhoria da qualidade de vidas das pessoas.
