# Supplementary material for: Ensino Médico como Interlocutor da Ciência e a Sociedade
Source: Arq Bras Cardiol. 2022 Nov 9;119(5 Suppl 1):4–5. [Article in Portuguese] doi: 10.36660/abc.20220491 (PMC9750194; doi:10.36660/abc.20220491)
Supplement: Translation

# Translation: Medical Education: Interlocutor of Science and Society

**Authors:** José Maria Peixoto

**Affiliations:** 1 Brazil Universidade José do Rosário Vellano, Campus Belo Horizonte, Belo Horizonte, MG – Brazil

**Keywords:** Education, Medical, Learning, Cardiology/education, Scientific Research and Technological Development, Faculty, Medicine

Scientific progress in Cardiology is unquestionable.^1 , 2^ We must have in mind, however, that the propelling of this development lies on *education sciences* , which transform the world by mobilizing people to knowledge. Despite the debate on what types of knowledge are related to them, according to Kant,^3^ education sciences enable individuals to reach autonomy and freedom to live in society.

Traditional science produces knowledge from experiment and observation, using rational methods that guarantee the validity of the results. Nevertheless, these assumptions favor the separation between the *subject* and the *object* of study, resulting in a technical-scientific culture that often ignores philosophical thinking, which drives individual’s desire for knowledge.^4^

Science for a long time has no longer been a method detached from social issues, but rather, has served the needs of the community.^5^ The COVID-19 pandemic, started in Brazil in 2020,^6^ exemplifies this and will contribute to accelerate this process. The pandemic posed challenges to scientific research, which was required to provide fast responses to halt the advance of the disease, bringing the academic world closer to society. Augusti^5^ supports that it is necessary to think about a science committed to social practices, that encourages the production of collective teaching and learnings.^5^

At this point it is important the contribution of education sciences as an interlocutor between scientific knowledge and philosophy, the latter contributing to determine the purposes. Therefore, the different functions of the educational process are highlighted, which should be dedicated to individual’s professionalization without neglecting individual’s growth as a *person* , in terms of affective, cultural, ethical and political dimensions.^7^

Thus, despite the importance of technical-scientifical knowledge in professional formation, it is not sufficient to meet the demands of the contemporary world. The 1988 Constitution establishes health as a universal right, and the Brazilian Unified Health System (Sistema Único de Saúde, *SUS* ) as the centerpiece of the health care model in the country. The SUS assumes a change from a predominantly biomedical perspective of health care to a model that incorporates a biopsychosocial perspective, interdisciplinarity, and shared responsibility.^8^ There are still challenges in incorporating these professional competencies, requiring a debate on education in health and the formation of teachers in all spheres of medical education.^8^

In general, a good part of the discussion about medical training concerns to the undergraduate period, with little debate about postgraduate study, usually carried out in hospitals and medical specialty societies. Considering the importance of “lifelong learning”, medical education is a topic that should be addressed in these institutions, to foster research and teacher professionalization.^9^

Just as medical professional activity should incorporate the best scientific evidence for decision making, instructional strategies developed by institutions responsible for medical training should also be guided by scientific studies. Cardiology societies in the whole world have established criteria for certification of specialties and work areas; however, part of them have been determined by specialist consensus and not by research in education. There is hence the need to widen the discussion on medical education to all the community involved in medical training programs.^10^

Medical education is an internationally renowned field of scientific investigation involving a large academic community and specialized peer-review journals.^11^ Confusion still exists regarding the terms “Medical Education” and “Continuing Education”. Medical education is a field of knowledge dedicated to developing studies on the teaching-learning process, while continuing education focuses on knowledge acquisition and update.^12^Medical societies are generally more involved in continuing education activities, as those developed in congresses, seminars, courses etc.

Despite their importance, publications on education have been rarely covered my specialized medical journals, but rather published in health education journals. Therefore, many of these publications have been concentrated in the undergraduate program, although professional formation extends for life.

Allred et al.^11^ quantified the number of publications on education in cardiology and the number of cardiologists participating in education research and estimated the priority level of medical education studies in cardiology journals. The results were disappointing – of 26 cardiology journals included and 6645 articles screened, only four were on education. Ten general medical journals and 15 medical education journals were included, and of 6810 articles screened, only seven addressed cardiology-specific education and all have been published in medical education journals. It is of note that none of the authors of these seven articles were trained cardiologists. Regarding the percentage of cardiologists dedicated to medical education research, only 2.3% (n=128) out of 5584 researchers were cardiologists. Finally, the authors also assessed the cardiology journal mission statements and found that, of 1036 total words included, the term “educational” appeared only once, in the European Heart Journal.

Data of the present study highlight the little attention paid to medical education by cardiology journals. However, both cardiology societies and institutions for cardiology training have promoted teaching activities, and thus many cardiologists are involved in educational activities. Probably there has been no incentive to the development and publication of studies on medical education. In fact, it is difficult to publish studies about this topic in medical journals, due to the low priority given to these subjects.

The Brazilian Cardiology Society (SBC) is a scientific entity involved in medical knowledge production in Brazil. SBC has been renowned for its high-quality productions and innovations over the years. Its group of collaborators is composed of highly experienced specialists and researchers, who, in general, develop teaching activities at undergraduate and postgraduate levels, in addition to healthcare work. It is worth pointing out the important educational activities carried out by SBC, such as the *Universidade do Coração* (the Heart University), the preparatory course for certification in cardiology, and the relevant works conducted by the Judging Committee for the Cardiologist Title.

Although “Medical Education” appears to be a new topic, probably successful educational activities have already been performed by many in their work settings and not shared with the academic community. Therefore, the development of a supplement focusing medical education in cardiology would be an opportunity for teachers and researchers to share their findings and thereby foster the growth of a new area of professional activity and knowledge for cardiologists, the Medical Education.

Once again, corroborating its mission for innovative and contemporary actions, the SBC, specifically by members of the scientific board and the editorial board of the Arquivos Brasileiros de Cardiologia, led by the Editor-in-Chief Dr. Carlos Eduardo Rochitte, takes a step forward in offering strong support to research about education in cardiology, which is embodied in this Supplement. The space for high-quality publications on education in cardiology is guaranteed in the ABC Cardiol.

Medical education encompasses a variety of themes that may be within the SBC journals’ scopes, like teaching of clinical reasoning, the physician-patient relationship, curriculum development, teaching-research integration, teaching-community integration, teacher development, learning theories, assessment of the teaching-learning process, among others. Researchers involved in medical education in cardiology will be able to share the results of their works. By doing so, it is expected to encourage studies on this subject and the formation of study groups in medical education in the SBC, and to contribute for professionalization of teaching and preceptorship in cardiology in Brazil.

To invest in education and teacher training is an excellent way to form highly qualified professionals, capable to understand and meet social needs, seeking to improve people’s quality of life.
